# On the Relationship between Diabetes and Obstructive Sleep Apnea: Evolution and Epigenetics

**DOI:** 10.3390/biomedicines10030668

**Published:** 2022-03-14

**Authors:** N. R. C. Wilson, Olivia J. Veatch, Steven M. Johnson

**Affiliations:** 1 Department of Microbiology and Molecular Biology, Brigham Young University, Provo, UT 84602, USA; nadzy.wilzod@gmail.com; 2 Department of Psychiatry & Behavioral Sciences, University of Kansas Medical Center, Kansas City, KS 66160, USA; oveatch@kumc.edu

**Keywords:** diabetes, obstructive sleep apnea, OSA, evolution, epigenetics

## Abstract

This review offers an overview of the relationship between diabetes, obstructive sleep apnea (OSA), obesity, and heart disease. It then addresses evidence that the traditional understanding of this relationship is incomplete or misleading. In the process, there is a brief discussion of the evolutionary rationale for the development and retention of OSA in light of blood sugar dysregulation, as an adaptive mechanism in response to environmental stressors, followed by a brief overview of the general concepts of epigenetics. Finally, this paper presents the results of a literature search on the epigenetic marks and changes in gene expression found in OSA and diabetes. (While some of these marks will also correlate with obesity and heart disease, that is beyond the scope of this project). We conclude with an exploration of alternative explanations for the etiology of these interlinking diseases.

## 1. Introduction

Diabetes is a condition characterized by hyperglycemia. Type I diabetes is generally regarded as an autoimmune disorder brought on by the body’s attack on the insulin producing cells of the pancreas. Type II diabetes is generally regarded as a progressive disease, with metabolic syndrome and insulin resistance giving way to full-blown type II diabetes, characterized by insufficient insulin production. Diabetes is associated with heart disease, some cancers, stroke, respiratory sensitivity, blurred thinking and dementia, kidney disease, nerve damage, blindness, depression, and death. Together, obstructive sleep apnea (OSA), diabetes, and cardiovascular disease make up a large proportion of the diseases of modern life. While we do not address causes of autoimmunity here, it should be noted that type I diabetes, along with allergies, arthritis, and other autoimmune disorders, is increasing alongside more obvious diseases of modern lifestyles, such as type II diabetes [1].

Diabetes is currently the seventh leading cause of death in the United States [2,3], with type II diabetes representing over 90% of all diabetes cases [4]. In addition, diabetes has been observed to either exacerbate symptoms of or increase risk for a number of the other top 10 causes of death in the United States over the last decade (e.g., heart disease [5], and cancer). Between 30 [5] and 80% [6] of diabetics (including type I diabetics) have comorbid OSA, and OSA is the most prevalent sleep disorder in the world [6]; OSA has been shown to be independently associated with insulin resistance, with the severity of OSA corresponding with the severity of insulin resistance [7,8]. OSA is also associated with gestational diabetes [9]. Sleep apnea is a condition in which breathing stops during sleep, either because the brain is not correctly signaling the body to breathe (central OSA, or CSA) or because soft tissues, primarily the tongue, collapse across the airway, obstructing it (obstructive sleep apnea, or OSA) [10]. OSA is associated with inflammation [8], metabolic syndrome and diabetes mellitus [11], obesity [12], heart disease [8], stroke [13], and death [14]. While obesity is one of the strongest risk factors influencing expression of both OSA and diabetes, these two conditions co-occur more often than can be explained by obesity alone [15]. A note on nomenclature: while insulin resistance is widely regarded as a major precursor for type II diabetes, type I diabetics can also experience insulin resistance. For the purposes of this review, we will be specific where sources allow, but, at times, the terms “diabetes,” “type II diabetes,” “metabolic syndrome,” and “insulin resistance” are used somewhat interchangeably.

This review aims to improve our understanding of the relationship between OSA and diabetes by exploring the shared evolutionary background of these diseases and examining epigenetic marks common to both conditions with a specific emphasis on exploring the non-traditional explanations of these diseases and their relationship. Improving our understanding of this relationship will lead to improved treatments for individuals with both conditions (e.g., reducing insulin resistance in patients with OSA). The field of Evo-Devo (evolutionary developmental biology) teaches us that evolutionarily successful changes usually target regulatory processes more often than underlying DNA sequences [10]. Thus, deciphering the epigenetic marks that are associated with diabetes and OSA should help illuminate the underlying etiology of these diseases and point to productive future avenues of research in the search for improved treatment and prevention of these major health issues.

## 2. The Generally Accepted Relationship between Obesity, Diabetes, and OSA and Their Cure

**Figure 1:**
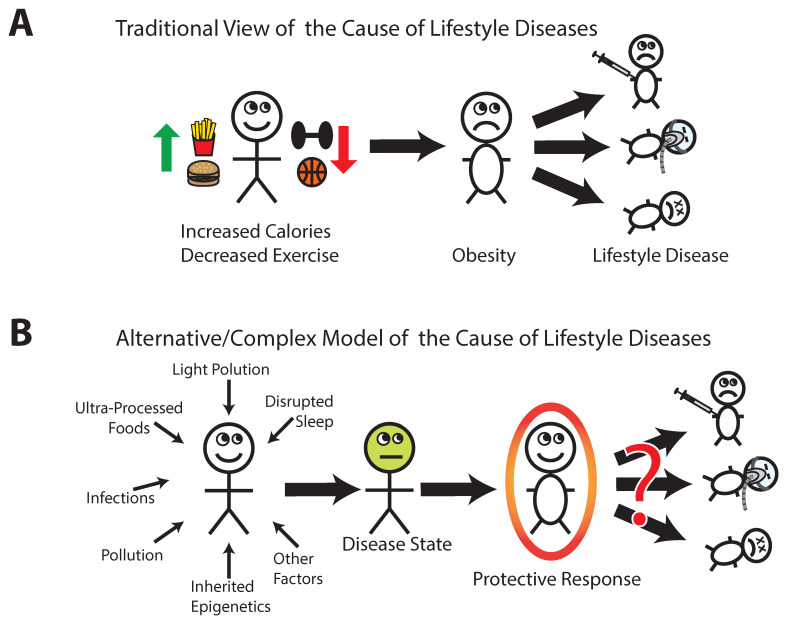
Models of the cause of lifestyle diseases: (**A**) a traditional model posits obesity as the lynchpin of lifestyle disease. If lifestyle diseases (diabetes, obstructive sleep apnea, etc.) are caused by obesity, and obesity is caused by eating too much and not exercising enough, the cure is simple and obvious. Unfortunately, human health and lifestyle diseases are generally much more complex than this model suggests; (**B**) an alternative model of lifestyle diseases. This model suggests that many factors interact to trigger a disease state in susceptible individuals, with obesity being a consequence of a combination of influences. Increased body mass index may be protective against the effects of many harmful factors, helping to delay or prevent negative health outcomes until a critical threshold has been reached where ongoing harmful inputs override protective benefits.

Widely held views of these diseases of modern life can be summarized in a series of statements outlined below (and Figure 1A), the assumed validity of which will be further analyzed:

- Prehistoric humans evolved during a time of food scarcity.
- Ingestion of too much fat and sugar results in obesity.
- The sole cause of obesity is eating too much and exercising too little.
- Obesity is a disease state with no benefit to the individual.
- Industrialization made fat, sugar, and calories in general widely available while making life easier, so humans do not have to expend as much energy in work and survival.
- The abundance of calories coupled with the lack of physical labor creates a mismatch between caloric intake vs. expenditure and results in obesity, diabetes, and heart disease.
- Sedentary individuals who eat too many calories are the people who get these diseases.
- Obesity causes OSA, diabetes, and heart disease.
- To cure these diseases, one would have to change human nature such that people would choose to eat less and exercise more.
- Individuals who exert themselves to eat less and exercise more can cure themselves of these diseases, including of obesity.

### 2.1. Prehistoric Humans May Not Have Evolved during a Time of Food Scarcity

There is a great deal of evidence to suggest that our distant ancestors had periods of prosperity, even to the point of allowing obesity [16]. In addition, it has been suggested that the agricultural revolution—in which humans undertook deliberate cultivation of food rather than the nomadic, gatherer-hunter lifestyle they had previously lived—led to various health issues such as anemia and loss of stature [17,18]. This seems an antithetical response for a species who had previously been underfed. Possibly the strongest evidence that our ancestors were not routinely deprived of calories, though, comes from the field of epigenetics. Many of the studies in this field focus on natural experiments, where humans were starved due to external factors such as war or famine [19]. Children and grandchildren of those underfed individuals show a strong predisposition toward obesity and heart disease, as well as diabetes and other metabolic problems. The fact that the epigenome is capable of changing with exposure to starvation conditions suggests that our ancestors had a non-starvation state available.

### 2.2. Ingestion of Too Much Fat and Sugar Is Not the Only Risk Factor for Obesity

From 1990s weight-loss guru Susan Powter’s mantra, “Fat makes you fat” to decades of FDA recommendations that Americans limit their refined sugar and fat consumption, we have all heard the message that fat and sugar are the biggest culprits in causing obesity and resultant diseases. Nina Teicholtz addresses this in her book, *The Big Fat Surprise* [20]. She looks at all the studies on which recommendations of low-fat diets have been made, and she presents the well-researched conclusion: we do not know what we think we know. Fat in our diets has not been shown harmful to our health and may well be helpful. Sugar is obliquely implicated in lifestyle diseases, but the data may not be as clear as previously accepted. What causes an individual to tip over from less than fit into downright unhealthy remains ill-defined. Both common sense and research suggest that there is a dietary component.

Reading Teicholtz, one also comes to the realization that some of the first nutritional researchers defined the boundaries of what we study regarding fats and sugars, and those boundaries have not been changed. It is hard to elucidate if some animal fats are better or safer than others (e.g., butter vs. lard) because the boundary of the study was drawn between animal and plant fats (e.g., butter and lard vs. canola oil and olive oil). The definitional boundaries having been drawn and unchallenged, all one can say for sure is that the effects of categories as currently defined are not well understood.

Fortunately, we do have some evidence for a dietary explanation of lifestyle diseases. A Brazilian nutrition researcher named Carlos Monteiro and his team were puzzled at data that showed people in Brazil were buying less sugar and less oil—but at the same time experiencing more obesity and more diabetes. Eventually, they reached the conclusion that the culprit was what they have labeled UPFs, or “Ultra Processed Foods” [21]. A well written summary of this with commentary can be found in Bee Wilson’s *How Ultra-processed Food Took Over Your Shopping Basket* [22]. While this classification has been opposed by some as “nonsensical”, it gained a great deal of traction when a physicist from the U.S. named Kevin Hall set out to show it was the content, rather than the processing, that was the problem. After some gold-standard pilot studies, Hall, himself, now suggests avoiding ultra-processed foods [23].

### 2.3. The Sole Cause of Obesity Is Not Eating Too Much and Exercising Too Little

This assumption is actually based on a truism, namely, if you put more into a system than goes out, the system must expand. It may expand gradually—as with obesity—or explode—as with the case of an overfilled water balloon—but expand it will. The problem with this particular application is that humans do not simply expand or contract according to how much they are holding. As living homeostatic systems, humans have numerous mechanisms for maintaining (among other things) temperature, hydration levels, electrolyte balance—and weight. 

In fact, however large or small one is, baseline metabolism accounts for the largest proportion of caloric expenditure, regardless of physiological overlay. This may be why exercise only slims a small part of the population: all humans benefit from exercise, but not all humans are tipped out of homeostasis into a more slender form by exertion.

Principles of biological homeostasis suggest that a healthy individual will not need to regulate their intake to the exact calorie on a given day—rather, the metabolism changes in mild ways to compensate for the inevitable caloric variability of diet—or, as expressed by St-Pierre and Tremblay:

“*The ability of healthy individuals to maintain body weight in the face of fluctuating energy intake and expenditure is due to an intricate physiological network that acts as the gatekeeper of energy balance. Indeed, under normal physiological conditions, any deviation in energy intake or expenditure is compensated for by physiological and behavioral responses that oppose these changes in order to return to a state of energy balance*” [24].

Additionally, unfortunate natural experiments such as the Dutch “hunger winter” [25] and alternating years of prosperity and famine in record-keeping Scandinavian countries [19] have given us the data to understand that low-calorie maternal (P0) conditions impel offspring (F1, F2, F3) to greater levels of many undesirable outcomes: obesity, metabolic syndrome, hypertension, type II diabetes, and other health conditions [26]. Metabolic syndrome was defined by the World Health Organization in 1998 as glucose intolerance and insulin resistance, either separately or together, in combination with two or more of the following: hypertension; elevated plasma triglycerides; reduced HDL cholesterol, central obesity, and microalbuminuria. Other definitions have been proposed, but the underlying concept of general metabolic disorder leading to further health troubles remains [27]. Modern data does not suggest that improving the caloric intake of these epigenetically affected offspring will reduce obesity (or other outcomes) in themselves (F1) or their offspring (F2, F3, etc.) [26].

### 2.4. Obesity May Be Beneficial When Concurrent with Type II Diabetes

As early as 1987, an entire issue of the *Journal of Obesity and Weight Regulation* was devoted to Ernsberger and Haskew’s alternative theories of obesity, namely, that it is not a disease state. They state clearly that obesity might result from a disease state and suggest that we may simply add to the problems of obesity if we try to “cure” it through the panacea of weight loss. Ernsberger and Haskew are not alone in their theories. There have been a number of articles published in the last few years bemoaning the “obesity paradox,” as it has been called—namely, that individuals who fall prey to diabetes or a number of other conditions live longer and have fewer complications than those who are slimmer when diagnosed [28,29]. Additionally, individuals who are “normal weight” have lower death rates than those who are merely 20% under “normal weight;” those who are above the “ideal weight” have lower death rates still [30].

Evolutionary biology teaches that conserved traits are generally important—and beneficial—to the group, and by extension, to the individual. The ability to become obese seems to be a conserved trait across species. As such, treating it as a disease state may be misleading and possibly even harmful. One explanation for the apparent paradox of obesity being closely associated with disease states, yet also being associated with better outcomes in those disease states, is that obesity could be protective against the true underlying causes of those health issues. The practice of removing adipose tissue through diet and surgery may not be providing the cures that are sought.

### 2.5. Numerous Environmental Risk Factors Increase Cross-Species Risk for Obesity

This is, in some ways, a reprise of the idea that humans are getting fatter because we are failing to properly account for our water-balloon-like nature in a changing environment. However, humans are not the only animals getting fatter. Such disparate species as wild marmosets and fully supervised lab rats have gotten bigger over the last few decades [27]. This suggests it is neither human character traits nor human diet and lifestyle choices that are causing the changes in human population fatness, but some other factor, be it light pollution or ultra-processed foods, or old-fashioned air pollution [31,32] or some other, unidentified cause; for example, in *Metabolic Implications of Body Fat Distribution*, Bjorntorp correlates several studies surrounding hormones, fat distribution, and insulin resistance. Bjorntorp declares, “this … suggests that endocrine aberrations may be of more importance than visceral fat accumulation for insulin resistance” [33]. Berreby covers most of these potential explanations for obesity in a well-written popular-press article [34].

It is important to note that throughout this paper, we use the term ‘fatness’ interchangeably with the term, ‘obesity’, although the first may be preferred, as being more precise. As Ernsberger and Haskew [30] point out, ‘obesity’ is defined by the mathematical derivation of body mass index (BMI), rather than actual body mass composition, whereas ‘fatness’ refers to the adipose content of the body. In deference to widespread convention, however, we have generally used the term ‘obesity’. Since BMI cannot distinguish between muscle mass and fat mass, unusual conditions such as sarcopenia would certainly skew the data driving these hypotheses, and further studies are needed to confirm or disprove any of these theories.

There is not adequate space here to explore the possibility of light pollution as a potential source of metabolic disruption but defer to Król and John R Speakman [35] and to Till Roenneberg for a more broad discussion on diurnal regulation [36].

### 2.6. Obesity May Be Protective against “Lifestyle Diseases”

While fatness is strongly associated with diseases of modern life, it may be the proverbial “healthy response to an unhealthy situation”—fatness may be protecting us from some of the worst effects of “lifestyle diseases” as we will discuss below.

### 2.7. Sedentary People Who Eat Too Many Calories Are Not the Only People Who Get These Diseases

Only half of obese people have metabolic syndrome, while fully 10% of the lean population has metabolic syndrome [37,38]. It is, of course, important to get this right: if obesity is the trigger for metabolic syndrome, with its concomitant heart disease and short path to diabetes, it would be logical—perhaps imperative—to target fatness as the first link in the chain for intervention. However, if it is not the first causative link in this chain, changing an individual’s body profile (from fatter to slimmer, for example) will at best do nothing to help their long-term health prospects and may even hurt them by interfering with an important response to an environmental stimulus. Even if weight loss does no harm per se, it may provide a false sense of progress against underlying disease, even while the underlying disease is still at work.

### 2.8. Obesity May Not Be the Primary Cause of OSA, Diabetes, and Heart Disease

Kapur [12] is a great example of how this reasoning has been perpetuated in academic writing: establishing from well-structured studies that OSA is correlated with weight gain, and that increased severity of OSA is correlated with increased weight gain, the author goes on to state that the weight gain clearly caused the OSA, though in the same paragraph he states that weight loss does not decrease severity of OSA to the same extent that weight gain increased it.

In a more supportable explanation of the connection between OSA and diabetes, Mansor et al. [39] asked why the hearts of diabetic patients often fail to repair themselves at the same rate as those of nondiabetic patients. They describe a metabolic shift that takes place when the cells of the heart need repair. Normally, hypoxic events cause a shift in heart-cell metabolism toward a high-glucose energy use. Furthermore, they discovered that this shift mechanism is still fully functional in a diabetic heart cell, but because the underlying diabetes shifts the cellular metabolism to a primarily fatty-acid-based energy use, the normal hypoxia-induced cascade does not lead to full glucose metabolism or normal cellular repair. Hypoxic events can be caused not only by heart attacks or strokes but also by temporary changes in breathing; and if those breathing changes occur during sleep, they are classified as sleep-disordered breathing, or OSA.

The field of linguistics holds other clues to support this possibility. The advent of fricatives in human speech corresponds with a softer diet, around the time of the first agricultural revolution [40]. Blasi et al. point out that softer foods can cause a difference in jaw development, leading to the modern human overbite and allowing fricatives such as “f” and “v” to enter human speech. Soft foods, such as grains (fermented and otherwise) and sweet fruit (more widely available when planted than when stumbled upon), would have been much more available after the first agricultural revolution than before. These soft foods are also much more likely to cause hyperglycemia, the primary diagnostic factor in diabetes.

Richard Wrangham [41] makes a strong case that *Homo habilis*, *Homo erectus*, and *Homo neanderthalensis* probably all cooked some of their food. That corresponds with smaller jaws and teeth in *Homo erectus* than in earlier hominids. Therefore, the final transition to having the ability to talk came with the final transition to being dependent on cooking in the most recent hominids, *Homo sapiens*.

Davidson et al. [42] hypothesized that obstructive OSA in humans is a side-effect of our capacity for speech. To evaluate this hypothesis, the authors evaluated men with OSA for severity of OSA and also measured several areas between the face and pharynx/larynx corresponding to the human capacity for speech. They found a strong correlation between some of these measurements (especially cranial base angulation and laryngeal descent) and the severity of the subjects’ OSA; this correlation corroborates the theory that OSA is a result of those same changes that allow speech.

Thus, this paleoarchaeological evidence in conjunction with modern research allows for the coevolution of OSA and hyperglycemia in response to a softer, cooked diet, although, of course, it does not prove it. However, there are further clues.

Evolutionary theory informs us that co-evolution indicates advantage to the organism—not as individuals but as a species. It is possible that our ancestors from 10,000 years ago got fat in conjunction with snoring. Anecdotal evidence suggests that we are much more likely to snore if sleeping alone or after physically demanding activities. This being the case, it has been postulated that snoring may serve as a last-ditch protection against predators who would risk going up against a sole human but not against a roaring crowd of humans—an impression of which may be given by some individual snorers. This could replace the theory of sleep-apnic snoring coevolving in conjunction with high carbohydrate diets, or it could stand beside it as additional adaptation.

### 2.9. Curing These Diseases May Not Require a Fundamental Change to Human Nature

Curing these diseases requires changing human nature if they are caused by innate human tendencies to overeat and under exercise. If, however, the diseases of modern life have a more complex pathogenesis involving interactions between personal choices and a host of environmental/epigenetic factors, it may be possible to stem the tide of lifestyle-based diseases by changing the environment. Importantly, even if only a small part of lifestyle-based diseases were caused by environmental/epigenetic factors, changing those factors could cure that portion of lifestyle-based diseases.

### 2.10. Eating Less and Exercising More May Not, of Itself, Effect a Cure—Even to Obesity

While there is much anecdotal evidence of long-term weight loss accompanied by a return to full health, a preliminary survey of the literature on weight loss and exercise interventions for treatment of diabetes revealed a very low long-term success rate. The most successful studies reported 5% body weight lost through non-surgical means, maintained for 3 years. Even surgical weight loss provided lowered dependence on (but not independence from) anti-diabetes drugs [43]. Other studies reported similar numbers over a similar time scale. While reduced calories and increased exercise appear to help ameliorate the health problems of modern life, they are not, of themselves, a cure.

## 3. The Relationship between OSA and Diabetes

### 3.1. A Brief Overview of Epigenetics

Epigenetics refers to the heritable changes in gene expression that occur without alterations to the underlying DNA sequence: biological inheritance transmitted outside the normal parameters of DNA. Epigenetic mechanisms regulate interactions between the genome and environmental factors such as infection and nutritional changes. The three primary epigenetic mechanisms currently being studied are DNA methylation, histone modifications, and noncoding RNAs. An excellent summary of DNA methylation and histone modifications can be found in Fodor, Cozma, and Karnieli [44]. Information on miRNAs can be found in Mishra, Zhong, and Kowluru [45].

Some epigenetic marks trigger other epigenetic marks: histone acetylation, for instance, can trigger a change in the methylation of the associated gene. Any disease that has genetic components that does not manifest until adulthood may be suspected of having an epigenetic component, or trigger. Diabetes, with its multitude of associated genes but unclear lines of inheritance, is a clear suspect for epigenetically triggered disease. OSA, also common in some ethnic groups but without clear inheritance patterns, may also have a strong epigenetic component.

### 3.2. If OSA and Diabetes Coevolved, We Should See Some Evidence

If OSA and diabetes coevolved, one would expect to see common epigenetic marks between the two conditions. To explore this idea, we undertook a review of extant literature regarding epigenetic marks and gene expression changes associated with diabetes and OSA. While many of the marks and genetic expression changes surveyed are unique to one of these conditions, there are several which appear in both of these conditions which do not appear in healthy controls. After cleaning the data and compiling it, we sorted it according to gene, protein/RNA expression, and associated disorder(s) and present the commonalities here.

### 3.3. Commonality of Epigenetic Marks for OSA and Diabetes (T2D)

There are numerous genes that encode proteins involved in the physiological response to insulin and oxygen levels where variation in epigenetic markers is evidenced to independently influence expression of genes and proteins in both T2D and OSA (Table 1). In the following section, the epigenetic mechanisms regulating a subset of these genes and the evidence supporting an evolutionary relationship between T2D and OSA are discussed. For a more comprehensive list of 214 identified genes, corresponding epigenetic markers and useful extracts, please see Supplemental Table S1.

#### 3.3.1. Genes Up- or Downregulated in Both OSA and T2D

The *AGT* gene encodes the angiotensin protein which acts as a vasoconstrictor helping to regulate blood pressure [60]. Angiotensin is also evidenced to be involved in the response to insulin; specifically, stimulation of the insulin receptor signaling pathway [61]. Furthermore, the angiotensin system may mediate the brain’s response to the oxygen containing compound estradiol which acts as a hyperactivator of astrocytes—in both males and females—in response to reduced oxygen levels in the brain [62,63]. Notably, the angiotensin gene promoter was observed to hypomethylated in the kidneys of T2D mouse models (db/db) [46]. In addition, an enhancer for *AGT* was observed to be hypomethylated in response to intermittent hypoxia, a typical manifestation of OSA, leading to increased expression of the angiotensin protein in neonatal mice [47]. As such, increased production of angiotensin may underlie the association between higher blood pressure and these two conditions. The question that then remains is how OSA and T2D induce this disruption of normal, homeostatic vasocontrol.

The *LEP* gene encodes leptin which is a key player in the regulation of energy balance and body weight control. Leptin is produced by specific types of adipose tissue and serves as a feedback regulator of fat storage, signaling satiety to the brain and also signaling the body to stop storing fat when there is enough [64]. The existence of “leptin-resistance,” akin to “insulin-resistance”, has been hypothesized on the basis that humans with high levels of leptin as well as large fat deposits appear to resist the hunger-damping signals of leptin [35]. It is evidenced to be involved in the response to hypoxia, as inferred from expression patterns in rats [65], and to insulin [66]. Increased methylation of the H4K20 histone modification in the leptin promoter region along with increased expression of the gene was observed in mice exposed to a high-fat diet in utero, suggesting that this histone modification might serve to enhance leptin expression [48,49]. Furthermore, reduced methylation of the gene has also been observed in mice exposed to a high-fat diet in late gestation [50]. This evidence from mouse models of diabetes is consistent with the reports of increased levels of leptin in the blood of individuals with OSA [51].

*MMP-9* encodes matrix metallopeptidase 9 which can be activated both directly and indirectly by reactive oxygen species [67], increased production of which are associated with OSA [68]. MMPs are important in breakdown of the extracellular matrix and are involved in such diverse processes as wound healing, learning, and memory. MMP-9 in humans has three fibronectin type II domains, which are collagen-binding domains, which is especially interesting given that several collagen-binding miRNAs are increased in diabetes. In diabetes, increased expression of the MMP-9 protein, as mediated by reduced methylation in the promoter, was observed in mice where diabetes was induced via streptozotocin administration [52]. In addition, increased acetylation of H3K9, which is a transcriptionally active mark, and decreased H3K9me2 has been observed in retinas of streptozotocin-induced diabetic rats and human donors [53]. Since H3K9me2 tends to repress transcription, this reduction is also consistent with increased expression of *MMP-9*. Consistent with a common etiology, Volna et al. found that increased levels of MMP-9 were positively correlated with hypoxemic severity indexes due to OSA (i.e., oxygen desaturation index (ODI) and sleep time spent at oxygen saturation below 90% (SpO2 < 90%)) in men suspected of OSA [55]. The over-expression of this protein could be the cause of slow diabetic wound healing and the brain fog associated with OSA, or a result of the body’s efforts to overcome those deficits, much as high insulin is generally a sign of poor insulin uptake (insulin resistance) rather than a cause of high blood sugars, of itself.

*SIRT1* encodes a class III histone deacetylase that has been shown to be downregulated with both OSA and diabetes [56,57,58,59]. Notably, the enzyme works to deacetylate hypoxia-inducible factor 1 alpha, effectively modulating cellular responses to hypoxia [69]. In addition, the enzyme has a possible regulatory effect on insulin-induced tyrosine phosphorylation of insulin receptor substrate 2, a vital step in the insulin signaling pathway [70]. Histone acetylation is generally an activating mark; deacetylation would be expected to reduce expression of the relevant gene. Conversely, a reduction in active deacetylases would be expected to result in upregulation of genes.

#### 3.3.2. The MicroRNAs Associated with OSA and T2D

In addition to epigenetic variation influencing gene expression in a consistent direction in both OSA and T2D, there are several microRNAs (miRNAs) associated with both conditions (Table 2) (for a more comprehensive list of 118 identified microRNAs with changes in either OSA or T2D, please see Supplemental Table S1). In general, miRNAs work to regulate gene expression via RNA silencing and posttranscriptional regulation indicating that upregulation of the miRNA would result in downregulation of the corresponding target. For example, miR-31 has been observed to be upregulated in relation to both diabetes and OSA [71,72]. Following miRNA expression profiling of streptozotocin-induced diabetic rat retinal epithelial cells, it was observed that miR-31 was significantly increased [72]. Similarly, in rat-derived cardiomyocytes it was observed that miR-31 was increased in response to chronic intermittent hypoxia in vitro [71]. Low levels of this miRNA are associated with gastric cancer invasion and metastasis [73]. Given that this miRNA is upregulated in both OSA and diabetes, in combination with the nutritional component of diabetes, the question is raised as to whether there is an evolutionary advantage conferred by diabetes. It is possible that a ubiquitous phenotype may generally be considered advantageous in some circumstances.

Another miRNA of interest identified via expression profiling of rat models of diabetes is miR-155 [72]. This miRNA is also observed in mice to be induced by hypoxia [74]. As miR-155 is a highly conserved master regulator of inflammatory diseases, including cancer and lung disease [77], it may confer protective effects against expression of diabetic neuropathies and lung disease in individuals with OSA.

Considering miRNAs function to regulate the expression of numerous genes, some miRNAs may be useful therapeutic targets. One of particular interest to diabetes and OSA is miR-146 which acts as a negative feedback regulator of *NF-κB*, observed to be elevated in both conditions [72,75]. *NF-κB* encodes a transcription factor that controls cytokine production and cell survival. It has been suggested that therapeutic targets of miR-146 may be useful to rescue the overexpression of *NF-κB* in individuals with diabetes to reduce inflammation [72]. An important direction for future research might be exploring the effects of miRNA-146 therapies to treat inflammation in OSA. Furthermore, TNF-α is another proinflammatory cytokine used by the immune system and released by macrophages as part of an inflammatory response to infection [78]. TNF-α is NF-κB-dependent and elevated in OSA [75]. Targeting an upstream mechanism to reduce *NF-κB* expression may also be beneficial to reducing overexpression of *TNF-α*.

Additional miRNAs of interest to potentially target for intervention to help reduce disease severity include miR-29c. Long et al. [76] found that miRNA-29c is upregulated in diabetes. In addition, miR-29c is almost perfectly complementary to the *Spry1* gene that produces the Sprouty homolog 1 protein. Furthermore, they established that knockdown of miRNA-29c by an antisense oligonucleotide has great promise in preventing kidney damage in diabetic patients. Zhang et al. [54] showed the upregulation of miR-21 with intermittent hypoxia. MiR-21 has been shown to be involved in arrhythmia and myocardial fibrosis; Spry1 is a downstream target of miR-21. The upregulation of miR-21 in OSA, therefore, provides a good window into the etiology of cardiac problems associated with OSA. The fact that both chronic intermittent hypoxia and high glucose levels induce changes in miRNA profiles associated with the Spry1/ERK/MMP-9 signaling pathway suggests a set of possible therapeutic targets with the potential to address health issues arising from both conditions.

## 4. Conclusions and Discussion

There are clearly a number of epigenetic marks common to OSA and diabetes which are not common to healthy controls. We focused here on DNA methylation, miRNAs, and histone modifications, but it is interesting to note that histone positioning and other epigenetic factors could also play a role in the pathogenesis of these conditions. Whether the two conditions arise from a common root cause, or whether they are two faces of the same condition, the possibility of coevolution has not been ruled out and remains an intriguing idea for further investigation.

### Some Possible Explanations for Coevolution of Lifestyle Diseases

One possible route to diabetes might go like this: light pollution disrupts the sleep cycle, throwing the immune system into difficulty. The chaotic immune system is unable to repair the microbiome, which is also under multiple assault from UPFs, lacking nutrients to properly build the body, and taking on preservatives which continue to do their job, even inside the body, namely: killing bacteria. As this multi-front assault proceeds, the body begins to pack on fat deposits, developing both leptin and insulin resistance in the process. This is a protective mechanism, protecting other body systems from metabolic assault. The poor nutrition from UPFs makes it harder for the body to produce insulin receptors and other needed proteins, hastening progression into full-blown metabolic syndrome. To offset some of the problems of metabolic syndrome, OSA begins to manifest. Eventually, the protective mechanisms of obesity and OSA fail, and blood teeters out of homeostatic equilibrium. Continued air pollution tips the scale into a spiral, landing in full-blown diabetes.

A second possibility: metabolic syndrome is triggered by some combination of overwork and undernutrition; OSA itself causes homeostatic glucose control to break, and the cascade begins. (While undernutrition often refers to a lack of calories, here it is used to indicate a lack of any essential nutrient, including fiber, minerals, and any other nutrient or vitamin necessary for healthy functioning.) Again, obesity is part of this cascade, but (again) as a protective measure. In this scenario, obesity is still a measure of poor health—but curing obesity will no more cure the underlying health problems than removing blood from the skin will cure a gut wound.

Another possible route: social stressors cause a constant assault of cortisol on body tissues, particularly heart tissue. The body triggers OSA to repair it. The OSA disturbs the immune system and thus the microbiome; the body, in an attempt to regulate and repair all this damage, starts storing up adipose tissue. In this scenario, UPFs then provide the perfect storm of poor fuel under external stressors to throw blood sugar regulation out the metaphorical window.

One last possibility: chemical exposure (possibly through air pollution) causes the body to pack on fat deposits, as a way to safely store those chemicals away from (literal) circulation. Those fat deposits might trigger leptin resistance, then insulin resistance, and so forth. Note that this is very similar to a conventional view of obesity and diabetes, outlined at the beginning of this paper, with the exception that it recognizes that not all causes of obesity are based on individual choices.

Other possible routes to metabolic syndrome can be envisioned, using the data available to us. Clearly, further rigorous research is needed. Whatever the case, solving this question—using real data rather than imagined scenarios—will lead to future research, understanding, and ultimately can change people’s lives.

## Figures and Tables

**Table 1:** Evidence of epigenetic modifications connecting OSA and T2D.

Gene	Associated Biological Process (Gene Ontology-Defined)	Associated Condition	Epigenetic Factors/Expression Differences
*AGT*	Response to oxygen-containing compound, response to insulin	Diabetic nephropathy	↓methylation in kidney of diabetic mice [46]
		OSA severity	↓methylation of enhancer, ↑gene expression in neonatal mice exposed to intermittent hypoxia [47]
*LEP*	Response to insulin, response to oxygen levels	Exposure to diet in utero	↑methylation and ↓acetylation of H4K20, ↓methylation of gene, ↑gene expression in mice exposed to high fat diet [48,49,50]
		OSA	↑protein levels in blood of OSA patients [51]
*MMP9*	Response to oxygen-containing compound	Diabetes/Diabetic retinopathy	↓methylation of promoter, ↑protein levels [52] ↑acetyl H3K9, ↓H3K9me2 in retinas of diabetic rats and humans [53]
		OSA severity	↑mRNA/protein in chronic intermittent hypoxia rats [54] ↑protein levels ≈ ↑ODI, ↑SpO2 < 90% in humans [55]
*SIRT1*	Response to insulin, response to oxygen levels	Diabetic retinopathy	↑miR-195, ↑miR-23b-3p, ↓gene expression in human and rat retinal cells exposed to high glucose [56,57]
		OSA	↓protein in humans with OSA [58,59]

**Table 2:** Evidence of microRNAs connecting OSA and T2D.

MicroRNA	Relevant Condition	Functional Evidence
miR-31	Diabetic retinopathy	Upregulated in diabetic rat retinal epithelial cells [72]
	OSA severity	Upregulation of miR-31 in response to chronic intermittent hypoxia [71]
miR-155	Diabetic retinopathy	Upregulated in diabetic rat retinal epithelial cells [72]
	OSA severity	Induced by hypoxia in mice [74]
miR-146	Diabetic retinopathy	Downregulates *NF-κB* which is overexpressed in diabetic rat retina [72]
	OSA	Downregulates *NF-κB* and *TNF-α* which are overexpressed in humans with OSA [75]
miR-29c and miR-21	Diabetic nephropathy	Upregulated in kidneys of diabetic mice [76]
	OSA severity	Upregulated in response to chronic intermittent hypoxia [54]

## Data Availability

Not applicable.

## Supplementary Materials

The following supporting information can be downloaded at: https://www.mdpi.com/article/10.3390/biomedicines10030668/s1, Supplemental Table S1 contains a list of all the genes, epigenetic marks, and non-coding RNAs evaluated in looking for commonalities between diabetes and sleep apnea. References [44,45,46,47,48,49,50,51,52,53,55,56,57,58,59,71,72,74,75,76,79,80,81,82,83,84,85,86,87,88,89,90,91,92,93,94,95,96,97,98,99,100,101,102,103,104,105,106,107,108,109,110,111,112,113,114,115,116,117,118,119,120,121,122,123,124,125,126,127,128,129,130,131,132,133,134,135,136,137,138,139,140,141,142,143,144,145,146,147,148,149,150,151,152,153,154,155,156,157,158,159,160,161,162,163,164,165,166,167,168] are cited in the supplementary material.
